# Predicting hyperkalemia in patients with advanced chronic kidney disease using the XGBoost model

**DOI:** 10.1186/s12882-023-03227-w

**Published:** 2023-06-12

**Authors:** Hsin-Hsiung Chang, Jung-Hsien Chiang, Chun-Chieh Tsai, Ping-Fang Chiu

**Affiliations:** 1 Division of Nephrology, Department of Internal Medicine, Antai Medical Care Corporation Antai Tian-Sheng Memorial Hospital, Pingtung County, Taiwan; 2 Department of Computer Science and Information Engineering, National Cheng Kung University, Tainan, Taiwan; 3 Division of Nephrology, Department of Internal Medicine, Changhua Christian Hospital, Changhua, Taiwan; 4 Department of Post Baccalaureate, College of Medicine, National Chung Hsing University, Taichung, Taiwan; 5 Department of Hospitality Management, MingDao University, Changhua, Taiwan

**Keywords:** Machine learning, Hyperkalemia, Chronic kidney disease

## Abstract

**Background:**

Hyperkalemia is a common complication of chronic kidney disease (CKD). Hyperkalemia is associated with mortality, CKD progression, hospitalization, and high healthcare costs in patients with CKD. We developed a machine learning model to predict hyperkalemia in patients with advanced CKD at an outpatient clinic.

**Methods:**

This retrospective study included 1,965 advanced CKD patients between January 1, 2010, and December 31, 2020 in Taiwan. We randomly divided all patients into the training (75%) and testing (25%) datasets. The primary outcome was to predict hyperkalemia (K^+^ > 5.5 mEq/L) in the next clinic vist. Two nephrologists were enrolled in a human-machine competition. The area under the receiver operating characteristic curves (AUCs), sensitivity, specificity, and accuracy were used to evaluate the performance of XGBoost and conventional logistic regression models with that of these physicians.

**Results:**

In a human-machine competition of hyperkalemia prediction, the AUC, PPV, and accuracy of the XGBoost model were 0.867 (95% confidence interval: 0.840–0.894), 0.700, and 0.933, which was significantly better than that of our clinicians. There were four variables that were chosen as high-ranking variables in XGBoost and logistic regression models, including hemoglobin, the serum potassium level in the previous visit, angiotensin receptor blocker use, and calcium polystyrene sulfonate use.

**Conclusions:**

The XGBoost model provided better predictive performance for hyperkalemia than physicians at the outpatient clinic.

**Supplementary Information:**

The online version contains supplementary material available at 10.1186/s12882-023-03227-w.

## Introduction

Hyperkalemia is a common complication of chronic kidney disease (CKD). The prevalence rate of hyperkalemia is approximately 9% in CKD patients and one-third of non-dialysis CKD patients under nephrology care [1, 2]. The risk factors for hyperkalemia in CKD patients are congestive heart failure, diabetes, old age, a high potassium diet, and medications like renin-angiotensin-aldosterone system inhibitors, beta-blockers, and others [3–5]. Hyperkalemia is associated with mortality, CKD progression, hospitalization, and high healthcare costs [1, 3].

There were some studies about predicting hyperkalemia. A claim study conducted in the U.S. successfully predicted hyperkalemia in CKD patients using logistic regression [6]. Several deep-learning models were performed well to predict hyperkalemia using electrocardiography in CKD patients and at the emergency department [7, 8].

Recently, machine learning have been developed to handle complex and high-dimensional data and increasingly applied in clinical medicine. The eXtreme Gradient Boost (XGBoost) algorithm developed by Chen et al. [9], one of the state-of-the-art gradient boosting machine learning algorithms, performed excellently in a number of medical problems [10–12]. In this study, we aimed to develop a machine learning model using the XGBoost algorithm and then assess the model performance in predicting hyperkalemia in patients with advanced CKD at the outpatient clinic in comparison to conventional logistic regression models and two nephrologists.

## Materials and methods

### Data source

This retrospective study used data retrieved from the pre-end-stage renal disease (pre-ESRD) program every 3 months that was initiated by Taiwan’s National Health Insurance Administration (NHIA) and performed in most of the hospitals in Taiwan to provide high-quality care for patients with CKD of stages 3b, 4, and 5 [13]. From January 1, 2010, to December 31, 2020, we used data collected in a single medical center in central Taiwan. This study was approved by the Institutional Review Board of Changhua Christian Hospital (IRB number-210423). All the data were measured in the laboratory that had been accredited by the College of American Pathologists’ Laboratory Accreditation Program.

### Study population

Eligible patients were enrolled to have had at least two outpatient visits in three months between January 1, 2010, and December 31, 2020. We excluded patients who were aged ≤ 20 years and whose estimated glomerular filtration rates (eGFRs) were ≥ 30 mL/min/1.73 m^2^ because advanced CKD patients with hyperkalemia had higher medical expenses and mortality rates [14, 15]. We also excluded patients who did not have serum potassium values in the *t*-th clinic visit or the *t + 1*-th clinic visit. The *t*-th clinic visit refers to the time when the lab tests were conducted for the development of the prediction models. We randomly divided the study participants into the training (~ 75%) and testing (~ 25%) datasets by patient identification to make sure that the data were totally different between the training and testing datasets.

### Model development

#### Predictors

**Fig. 1:**
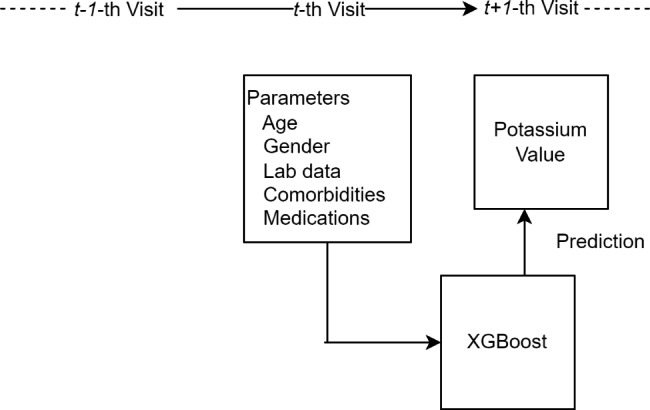
Model development and prediction of a single visit

Figure 1 shows how to generate parameters used for model development and prediction. The variables for our model consisted of demographics, laboratory tests, medical history based on ICD-9 and ICD-10 (Supplementary Table S1), and medications (Supplementary Table S2). For laboratory tests, missing values were imputed separately for the training and testing sets. We imputed the missing values using the K-Nearest Neighbors approach [16].

The primary outcome of our study was to predict whether or not hyperkalemia (K > 5.5 mEq/L) would occur during the *t + 1*-th visit.

#### Prediction machine learning algorithms

We built a binary prediction model using XGBoost and used a grid search with tenfold cross-validation to find the best hyperparameters. XGBoost is one of the ensemble decision-tree-based learning algorithms based on a gradient descent-boosting process. The core concept of gradient boosting decision tree algorithm is that it iteratively generates many weak classifiers and combines them to obtain a strong classifier, which is implemented by each new decision-tree learning from the errors of the previous decision-tree sequentially [17]. Other advantages of XGBoost are tuning hyperparameters, controlling overfitting, and parallel computation to reduce processing time [9, 12].

### Human-machine competition

Two nephrologists participated in our study. They predicted whether or not hyperkalemia would occur in the *t + 1*-th clinic visit using the data of the *t*-th clinic visit. We assessed their performance using the testing dataset and compared their results with those of XGBoost and the logistic regression model.

### Statistical analyses

We compared baseline characteristics between training and testing datasets. Categorical variables were presented as proportions and continuous variables were presented as mean values with standard deviations. Numerical variables of clinical characteristics were compared using Student’s *t*-test. The chi-squared test was used to compare differences in categorical variables.

We conducted multivariable logistic regression analyses as a reference model. The overall performance of the models in the testing dataset was assessed by calculating the area under the receiver operating characteristic curve (AUC) and the associated 95% confidence interval (CI). The AUC values were compared using the DeLong test. The net benefit of the XGBoost model was assessed using the decision curve analysis (DCA) and then further using clinical impact curves (CIC) to assess the clinical practicability [18, 19]. Sensitivity, specificity, positive predictive value (PPV), negative predictive value (NPV), and accuracy were calculated to evaluate the model performance. Finally, we used the SHAP (SHapley Additive exPlanations) framework to evaluate the impact of features in our model [20].

Machine learning algorithms and statistical analyses were performed using Python version 3.9.12, scikit-learn version 1.0.2, and R version 4.2.0.

## Results

### General demographics

**Fig. 2:**
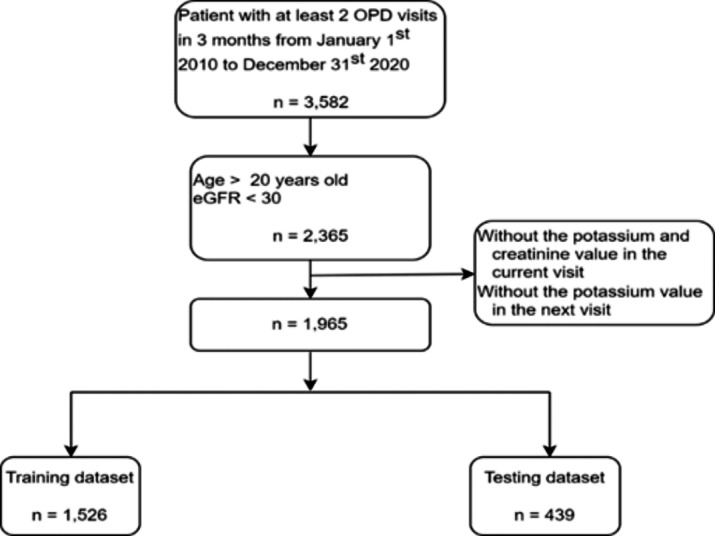
Participant flow diagram. *Abbreviations*: OPD, outpatient department

The 1,526 patients (6,949 visit numbers) in the training dataset and 439 patients (2,054 visit numbers) in the testing dataset met our inclusion criteria (Fig. 2). Baseline patient characteristics are presented in Table 1. The mean patient age was 69.39 years and 49.2% were female. Patients in the testing dataset were older and more likely to be female, have diabetes, cardiovascular disease, cancer, hypertension, hyperlipidemia, and dementia. Patients in the testing dataset had a higher proportion of prescriptions for angiotensin receptor blocker and lower proportion of calcium polystyrene sulfonate use. The prevalence of hyperkalemia (K > 5.5 mEq/L) during the *t + 1*-th visit was 6.6% in the training dataset and 6.8% in the testing dataset.

**Table 1:** Baseline characteristics in the training and testing datasets

	All	Training set	Testing set	P value
N (total visit numbers)	9003	6949	2054	
Age (year)	69.39 ± 12.99	68.24 ± 13.02	73.26 ± 12.14	< 0.001
Gender (female)	4430 (49.2%)	3229 (46.5%)	1201 (58.5%)	< 0.001
Laboratory data				
Hemoglobin (g/dL)	10.47 ± 1.75	10.49 ± 1.76	10.38 ± 1.73	0.012
Albumin (g/dL)	3.66 ± 0.42	3.67 ± 0.43	3.65 ± 0.40	0.153
Creatinine (mg/dL)	4.15 ± 2.33	4.24 ± 2.38	3.83 ± 2.13	< 0.001
BUN (mg/dL)	53.17 ± 25.71	54.01 ± 26.14	50.33 ± 24.00	< 0.001
Uric acid (mg/dL)	6.66 ± 1.82	6.66 ± 1.83	6.63 ± 1.76	0.459
Sodium (mEq/L)	137.57 ± 13.02	137.45 ± 3.75	137.98 ± 26.37	0.109
Potassium (mEq/L)	4.52 ± 0.63	4.52 ± 0.63	4.51 ± 0.63	0.544
Calcium (mg/dL)	8.99 ± 0.61	8.98 ± 0.60	9.03 ± 0.63	0.003
Phosphate (mg/dL)	4.27 ± 0.96	4.29 ± 0.97	4.19 ± 0.92	< 0.001
Comorbidity				
CAD	1837 (20.4%)	1326 (19.1%)	511 (24.9%)	< 0.001
Cancer	1182 (13.1%)	883 (12.7%)	299 (14.6%)	0.032
CHF	4968 (55.2%)	3838 (55.2%)	1130 (55.0%)	0.882
CVA	1379 (15.3%)	949 (13.7%)	430 (20.9%)	< 0.001
Dementia	321 (3.6%)	211 (3.0%)	110 (5.4%)	< 0.001
Diabetes	4467 (49.6%)	3365 (48.4%)	1102 (53.7%)	< 0.001
Dyslipidemia	4947 (54.9%)	3694 (53.2%)	1253 (61.0%)	< 0.001
Hypertension	7198 (80.0%)	5504 (79.2%)	1694 (82.5%)	0.001
Medications				
ACEi	350 (3.9%)	260 (3.7%)	90 (4.4%)	0.21
ARB	4484 (49.8%)	3388 (48.8%)	1096 (53.4%)	< 0.001
CCB	5444 (60.5%)	4168 (60.0%)	1276 (62.1%)	0.086
Beta-blocker	2259 (25.1%)	1736 (25.0%)	523 (25.5%)	0.68
Alpha-/beta-blocker	1020 (11.3%)	775 (11.2%)	245 (11.9%)	0.35
Direct vasodilator	125 (1.4%)	100 (1.4%)	25 (1.2%)	0.517
Potassium-sparing diuretic	176 (2.0%)	143 (2.1%)	33 (1.6%)	0.227
Loop diuretic	2466 (27.4%)	1891 (27.2%)	575 (28.0%)	0.503
Corticosterioid	1046 (11.6%)	816 (11.7%)	230 (11.2%)	0.523
Anticoagulant	163 (1.8%)	133 (1.9%)	30 (1.5%)	0.208
Antiplatelet	2019 (22.4%)	1510 (21.7%)	509 (24.8%)	0.004
ESA	2646 (29.4%)	2063 (29.7%)	583 (28.4%)	0.266
CPS	1105 (12.3%)	889 (12.8%)	216 (10.5%)	0.006
NSAID	274 (3.0%)	188 (2.7%)	86 (4.2%)	0.001
Proton-pump inhibitor	1126 (12.5%)	851 (12.2%)	275 (13.4%)	0.181
Statins	3776 (41.9%)	2886 (41.5%)	890 (43.3%)	0.154
Oral hypoglycemic	2916 (32.4%)	2225 (32.0%)	691 (33.6%)	0.176
Insulin	1522 (16.9%)	1178 (17.0%)	344 (16.7%)	0.854
Xanthine oxidase inhibitor	4588 (51.0%)	3485 (50.2%)	1103 (53.7%)	0.005
Hyperkalemia episode (K > 5.5 mEq/L) in the t + 1-th clinic visit	601 (6.6%)	460 (6.6%)	141 (6.8%)	0.733

*Abbreviations*: CHF, congestive heart failure; CAD, coronary artery disease; CVA, Cerebrovascular accident; ACEi, Angiotensin-converting enzyme inhibitors; ARB, Angiotensin receptor blocker; CCB, Calcium channel blocker; ESA, Erythropoiesis stimulating agent; CPS, Calcium polystyrene sulfonate; NSAID, Nonsteroidal anti-inflammatory drug

### Development of the XGBoost Model and comparison of human-machine competition

**Fig. 3:**
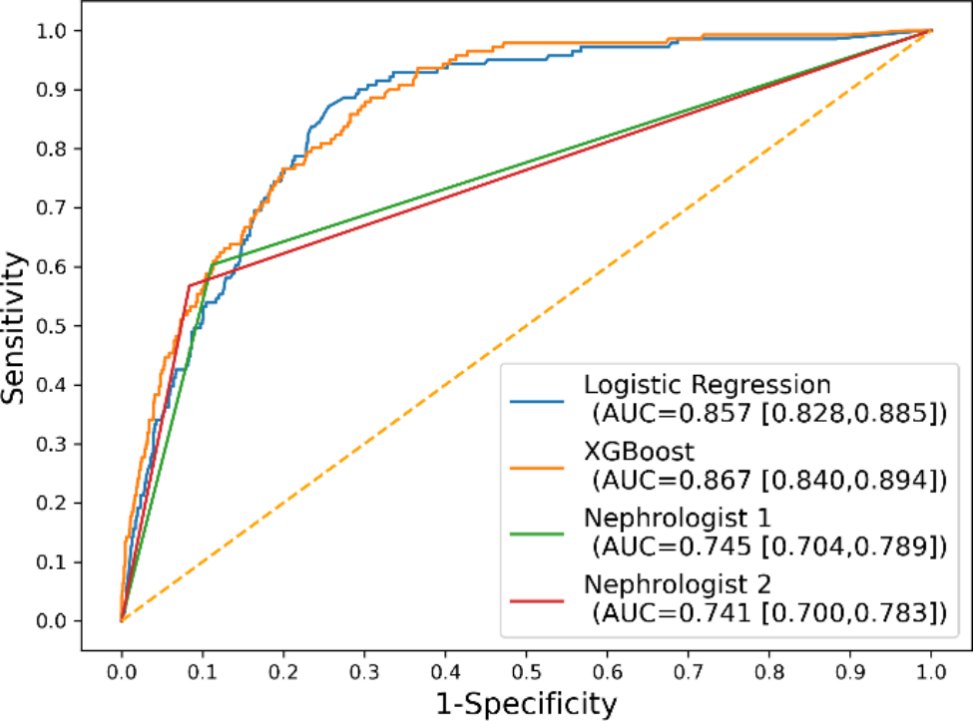
Area under operating characteristic (ROC) curves for XGBoost, logistic regression models, and nephrologists in the testing dataset

**Fig. 4:**
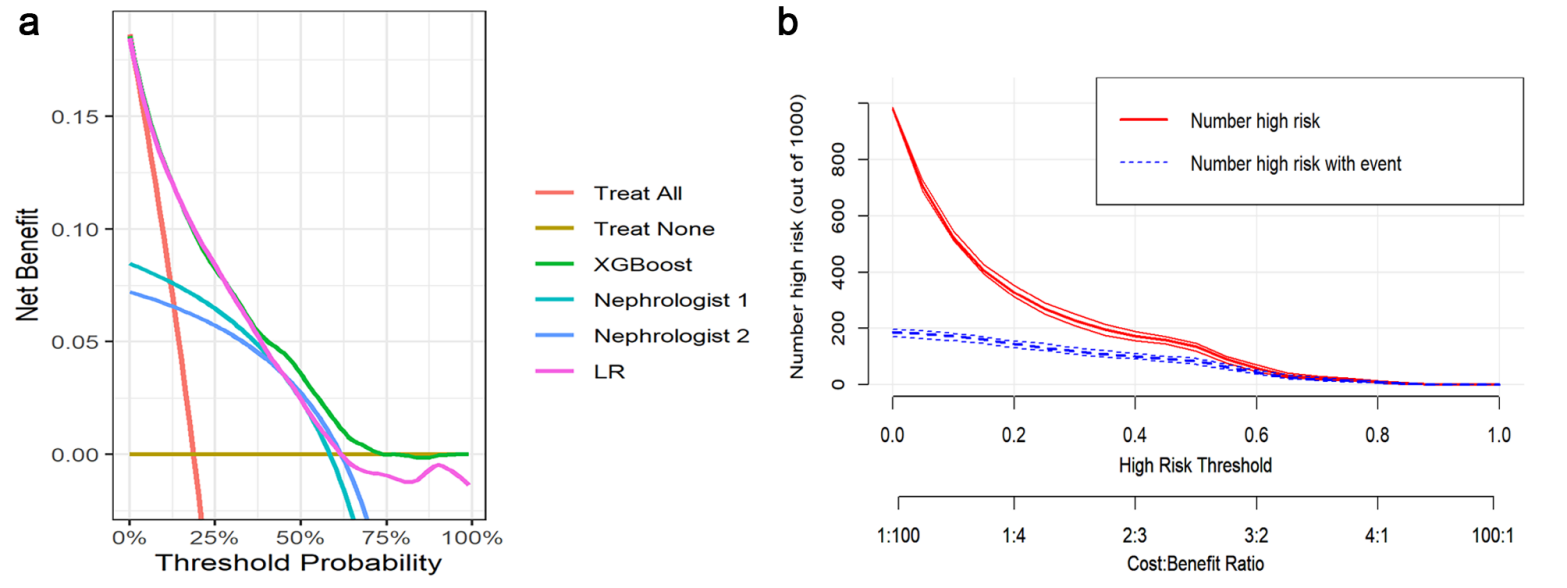
**A**. Decision curve analysis (DCA) of the XGBoost, logistic regression (LR) models and nephrologists. XGBoost and LR models demonstrated a larger net benefit compared to nephrologists for the threshold probabilities. **B**. Clinical impact curve (CIC) of the XGBoost model

The detailed results of the human-machine competition are shown in Table 2 and Fig. 3. In detecting hyperkalemia, the XGBoost model had the highest AUC, PPV, and accuracy in the human-machine competition. In terms of the AUC, the performance of the XGBoost model was significantly better than that of the two clinicians (0.867, 95% CI 0.840–0.894, vs. 0.745, 95% CI 0.704–0.789, and 0.741, 95% CI 0.700–0.783, respectively); however, its performance did not differ significantly from that of logistic regression. The net benefit for the XGBoost and logistic regression models was better than that of the two clinicians based on DCA (Fig. 4a). Figure 4b shows the XGBoost model also had a better clinical net benefit within a wide range of threshold probabilities and impacted patient outcomes.

### Important features of the XGBoost model and results of multivariable logistic regression

**Fig. 5:**
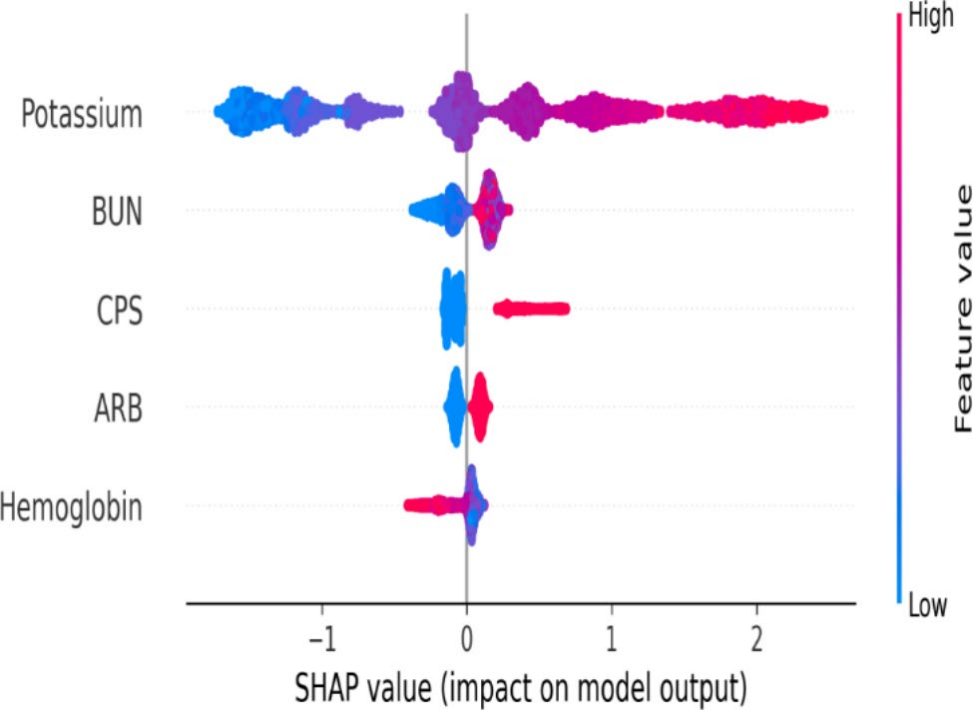
Top 5 important features of the XGBoost model by SHAP value

As shown in Fig. 5, the top 5 features of the XGBoost model were the potassium level during the t-*th* visit, *blood urea nitrogen*, calcium polystyrene sulfonate, angiotensin receptor blocker use, and hemoglobin, in that order. Supplementary Table 3 shows the results of univariate and multivariate logistic regression analysis. In the multivariate logistic regression analysis, the top 5 significant variables by *P* value for hyperkalemia were potassium level during the t-*th* visit (OR, 6.96; 95% CI, 6.05–8.02; P < 0.001), ARB (OR, 1.40; 95% CI, 1.19–1.64; P < 0.001), hemoglobin (odds ratio [OR], 0.92; 95% [CI], 0.87–0.97; P = 0.001), CHF (OR, 0.8; 95% CI, 0.68–0.95; P = 0.008), and calcium polystyrene sulfonate (OR, 1.29; 95% CI, 1.06–1.56; P = 0.009) (Table 3).

**Table 2:** Performance comparison between XGBoost model, logistic regression model, and nephrologists in the training and testing datasets

Training dataset				
Model	XGBoost	LR		
AUC (10-fold)	0.827	0.816		
95% CI of AUC	0.808–0.845	0.798–0.835		
Sensitivity	0.041	0.013		
Specificity	0.996	0.996		
PPV	0.441	0.222		
NPV	0.936	0.934		
ACC	0.933	0.931		
Testing dataset				
Model	XGBoost	LR	Nephrologist 1	Nephrologist 2
AUC	0.867	0.856	0.745	0.741
95% CI of AUC	0.840–0.894	0.828–0.885	0.704–0.789	0.700-0.783
Sensitivity	0.049	0.014	0.602	0.567
Specificity	0.998	0.998	0.888	0.916
PPV	0.700	0.500	0.285	0.333
NPV	0.934	0.932	0.968	0.966
ACC	0.933	0.931	0.869	0.892

*Abbreviations*: LR, logistic regression, PPV, positive predicted value; NPV, negative predicted value; ACC, accuracy

**Table 3:** The top 5 significant variables by *P* value in the multivariate logistic regression model

Significant variables	Odds Ratio (95% CI)	P value
Potassium level in the t-th visit	6.96 (6.05–8.02)	<0.001
ARB	1.40 (1.19–1.64)	< 0.001
Hemoglobin	0.92 (0.87–0.97)	0.001
CHF	0.80 (0.68–0.95)	0.008
Calcium polystyrene sulfonate	1.29 (1.06–1.56)	0.009

*Abbreviations: ARB*, Angiotensin receptor blocker; CHF, congestive heart failure

## Discussion

In the present study, we developed the XGBoost model to predict hyperkalemia in advanced CKD patients using data from an outpatient clinic. The XGBoost model demonstrated better performance in comparison with two nephrologists; however, the difference in AUC between XGBoost and the logistic models was not statistically significant.

The prevalence and incidence rates of ESRD in Taiwan are the highest in the world [21, 22]. Taiwan’s NHIA developed the pre-ESRD program to reduce the magnitude of the problem of CKD in 2006; as such, nephrologists may often need to attend to more than 20 CKD patients at a clinic. Clinical decision-making tools could help physicians make better decisions in properly caring for patients in Taiwan, especially when they face many CKD patients at a clinic. Hyperkalemia is a frequent complication of CKD due to its limited ability to increase potassium excretion [4, 23]. Hyperkalemia is associated with not only muscle weakness and fatal arrhythmia but also high insurance costs in CKD patients [1, 24]. Thus, we investigated whether the XGBoost model improved hyperkalemia prediction for CKD patients. The XGBoost model performed best in human-machine competition using evaluation metrics such as the AUC, accuracy, NPV, and PPV in this study. In addition, the XGBoost model had a higher bet benefit than the logistic regression model, which would lead to the better clinical outcomes [25].

XGBoost is an efficient and flexible gradient boosting machine learning algorithm and make prediction well in clinical problems. XGBoost achieved a high accuracy in predicting COVID-19 severity in US, excellently predicted kidney outcome in immunoglobulin A nephropathy, and outperformed 2-year dementia risk [11, 26, 27]. In this study, The XGBoost model performed best. However, the differences in evaluation metrics between the XGBoost and logistic regression model were not statistically significant. Evidence has revealed that logistic regression was not inferior to machine learning for clinical prediction models [28]. The possible reason why machine learning does not perform better in clinical problems is the fact that clinical predictions have a poor signal-to-noise ratio, low-dimensional data, and a small sample size [28, 29].

Machine learning and logistic regression usually use different variables with divergent ranks to develop prediction models [29]. In addition, machine learning models are regarded as black-box models so that physicians may doubt the results [30]. In this study, we attempted to explore if the XGBoost could use reasonable variables to develop a prediction model. We used SHAP to visualize the five most important features in the XGBoost model and compared the results to that of the logistic regression model. In both models, there were four variables that were chosen as high-ranking variables, including hemoglobin, the serum potassium value during the t-th visit, angiotensin receptor blocker use, and calcium polystyrene sulfonate use. A high potassium value during the t-th visit and calcium polystyrene sulfonate use implies that the baseline potassium level of patients is high. Angiotensin receptor blocker use induced hyperkalemia due to the decline in the serum aldosterone level and decrease in the renal blood flow [31, 32]. Lower hemoglobin levels were associated with hyperkalemia, and possible risk factors include iron-deficiency anemia, sickle cell anemia [33], and gastrointestinal bleeding [34]. From the above results, we believe that the XGBoost algorithm developed a reliable prediction model using the variables that have clinical significance in this study.

We may develop a clinical decision support system which has reasonable clinical performance to help physicians identify high-risk patients with hyperkalemia. The system would alarm the CKD team that patients are in danger of hyperkalemia so that they can prescribe medications to prevent hyperkalemia and inform patients of going back to the clinic for follow-up earlier under the care of multidisciplinary teams. Nevertheless, there are some limitations to the present study. First, this is a single-center study and it may not be able to apply to other hospitals directly (absent external validation). Second, this dataset did not include vital signs, blood gas data, oral sodium bicarbonate, body weights, other nutritional parameters, lifestyles, and physical statuses, all of which may affect the potassium level. Third, the data of the pre-ESRD program in Taiwan were collected every 3 months. We are not able to retrieve the data if patients have data between 2 clinic visits within 3 months. Finally, there were missing values in this dataset; thus, a prospective study in which complete data can be collected is recommended to verify our findings.

In conclusion, the XGBoost model had a better predictive performance for hyperkalemia than physicians in an outpatient clinic. The results indicate that this model may be a decision-making tool to help physicians take better care of patients. Further prospective studies are needed to validate our findings.

## Electronic supplementary material

Below is the link to the electronic supplementary material.

**Supplementary Materials: Table S1**. ICD-9 and ICD-10 diagnostic codes used to identify comorbidities. **Table S2**. Medications used in this study. **Table S3**. Logistic regression analyses yielding odds ratios for factors associated with hyperkalemia in patients with advanced chronic kidney disease

## Data Availability

The dataset supporting the conclusions of this article is included within the article.

## Abbreviations

CHF: congestive heart failure
CAD: coronary artery disease
CVA: Cerebrovascular accident
ACEi: Angiotensin-converting enzyme inhibitors
ARB: Angiotensin receptor blocker
CCB: Calcium channel blocker
ESA: Erythropoiesis stimulating agent
CPS: Calcium polystyrene sulfonate
NSAID: Nonsteroidal anti-inflammatory drug
CKD: Chronic kidney disease
LR: Logistic regression
PPV: Positive predicted value
NPV: Negative predicted value
ACC: Accuracy
BUN: blood urea nitrogen
CIC: Clinical impact curve
DCA: Decision curve analysis
