# Supplementary material for: Predicting hyperkalemia in patients with advanced chronic kidney disease using the XGBoost model
Source: BMC Nephrol. 2023 Jun 12;24:169. doi: 10.1186/s12882-023-03227-w (PMC10259360; doi:10.1186/s12882-023-03227-w)
Supplement: Supplementary file 1 — Supplementary Materials: Table S1. ICD-9 and ICD-10 diagnostic codes used to identify comorbidities. Table S2. Medications used in this study. Table S3. Logistic regression analyses yielding odds ratios for factors associated with hyperkalemia in patients with advanced chronic kidney disease [file 12882_2023_3227_MOESM1_ESM.docx]

Supplementary Materials

Table S1. ICD-9 and ICD-10 diagnostic codes used to identify comorbidities.

| Diagnosis | ICD-9 diagnosis codes | ICD-10 diagnosis codes |
| --- | --- | --- |
| Coronary artery disease | 410.x, 411.x, 412.x, 413.x, 414.x | I20-I25 |
| Cancer | Between '196.0' and '199.1', between '209.70' and '209.75', '209.79', '789.51' / Metastatic cancer / | C81, C83.3, C82–C85, and C91 |
|  | Between '200.00' and '202.38', between '202.50' and '203.01', between '203.02' and '203.82', '238.6', '273.3' / Lymphoma / | Buccal cavity and pharynx (C00-C14; C462), digestive organs (C15–C26), respiratory system including thoracic organs (C30–39; C450), bones, joints and articular cartilage (C40–C41), skin (C43–C44; C460), mesothelium and connective tissue (C451–C459; C461; C463; C467; C468; C469; C47–C49; B210), breast (C50), female genital organs (C51–C58), male genital organs (C60–C63), urinary tract (C64–C68; D090–D091; D301–D309; D411–D419), eye and central nervous system (C69–C72; C751–C753; D32–D33; D352–D354; D42–D43; D443–D445), and endocrine glands (C73–C74; C750; C754–C759). |
|  | Between '140.0 ' and '172.9', between '174.0 ', between '258.01' and '258.03', between '209.00' and '209.24', between '209.25' and '209.30', '175.9', '179', '195.8', '209.30', '209.36' / Solid tumor without metastasis / |  |
| Congestive heart failure | 425.x; 428.x; 404.01, 404.03, 404.11, 404.13, 404.91, 404.93, 398.91, 402.01, 402.11, 402.91 | I50.0-I50.9 |
| Cerebrovascular accident | 430-438 | I60-I69 |
| Dementia | 290.0, 290.1, 290.2, 290.3, 290.4, 294.x, 331.0, 331.1, 331.5, 331.82 | F00.x, F01.x, F02.x, F03.x, G30.x |
| Diabetes | 249', '250' | E08-E13 |
| Dyslipidemia | 272.0, 272.1, 272.2, 272.3 or 272.4 | E78 |
| Hypertension | '401', '402', '403', '404', '405' | I10–I13 |

Table S2. Medications used in this study

| Drugs classification | Brand names |
| --- | --- |
| Angiotensin-converting enzyme (ACE) inhibitor | Lisinopril, Captopril, Imidapril |
| Potassium sparing diuretic | Spironolactone |
| Adrenal Corticosteroid | Prednisolone, Methylprednisolone |
| Alpha- and beta-adrenergic blocker | Carvedilol, Labetalol |
| Anticoagulant | Warfarin, Rivaroxaban, Apixaban, Dabigatran |
| Antiplatelet | Aspirin, Clopidogrel |
| Angiotensin receptor blocker | Valsartan, Telmisartan, Irbesartan, Olmesartan, Losartan, Candesartan |
| Direct vasodilator | Hydralazine |
| Calcium channel blocker | Nifedipine OROS, Amlodipine, Servidipine, Lercanidipine |
| Erythropoiesis stimulating agent | Methoxy polyethylene glycol-epoetin beta, Epoetin beta |
| Calcium polystyrene sulfonate | Kayexalate |
| Oral hypoglycemic | Pioglitazone, Vildagliptin, Glimepiride, Saxagliptin, Linagliptin, Sitagliptin, Repaglinide |
| Nonsteroidal anti-inflammatory drug | Etoricoxib, Diclofenac, Celecoxib, Sulindac |
| Proton Pump Inhibitor | Esomeprazole, Dexlansoprazole, Lansoprazole |
| Phosphodiesterase inhibitor | Pentoxifylline |
| Beta Blocker | Bisoprolol, Propranolol, Atenolol |
| Loop diuretic | Furosemide |
| HMG-CoA reductase inhibitor | Fluvastatin, Rosuvastatin, Atorvastatin, Pitavastatin, Pravastatin, |
| Xanthine oxidase inhibitor | Allopurinol, Febuxostat |
| Insulin | Humalog, Novomix, NovoRapid, Insulin glargine |

Table S3. Logistic regression analyses yielding odds ratios for factors associated with hyperkalemia in patients with advanced chronic kidney disease

| Variable | Univariate Analysis | | Multivariate Analysis | |
| --- | --- | --- | --- | --- |
|  | OR (95% CI) | *P* Value | OR (95% CI) | *P* Value |
| Potassium | 7.99 (7.00-9.15) | <0.001 | 6.96 (6.05-8.02) | <0.001 |
| ARB | 1.81 (1.61-2.05) | <0.001 | 1.40 (1.19-1.64) | <0.001 |
| Hemoglobin | 0.85 (0.82-0.89) | <0.001 | 0.92 (0.87-0.97) | 0.001 |
| CHF | 0.94 (0.83-1.06) | 0.278 | 0.80 (0.68-0.95) | 0.008 |
| CPS | 2.46 (2.11-2.88) | <0.001 | 1.29 (1.06-1.56) | 0.009 |
| Beta-blocker | 1.16 (1.01-1.33) | 0.033 | 1.22 (1.03-1.44) | 0.021 |
| ACEI | 1.47 (1.10-1.95) | 0.009 | 1.44 (1.01-2.03) | 0.039 |
| Female | 0.89 (0.79-1.00) | 0.06 | 0.85 (0.73-1.00) | 0.047 |
| Age | 1.00 (0.99-1.00) | 0.083 | 1.00 (1.00-1.01) | 0.684 |
| Albumin | 0.88 (0.76-1.01) | 0.062 | 1.01 (0.84-1.22) | 0.906 |
| BUN | 1.01 (1.01-1.01) | <0.001 | 1.00 (1.00-1.01) | 0.275 |
| Uric acid | 1.03 (0.99-1.06) | 0.131 | 1.01 (0.97-1.05) | 0.689 |
| Sodium | 0.99 (0.97-1.00) | 0.108 | 1.01 (0.99-1.03) | 0.622 |
| Calcium | 0.91 (0.82-1.01) | 0.069 | 0.96 (0.84-1.10) | 0.549 |
| Phosphate | 1.32 (1.24-1.39) | <0.001 | 0.97 (0.88-1.07) | 0.492 |
| CAD | 1.01 (0.87-1.18) | 0.891 | 1.05 (0.87-1.27) | 0.602 |
| Cancer | 0.88 (0.73-1.06) | 0.186 | 1.03 (0.83-1.28) | 0.772 |
| CVA | 0.83 (0.69-1.00) | 0.053 | 0.90 (0.72-1.11) | 0.324 |
| Dementia | 0.58 (0.37-0.86) | 0.01 | 0.65 (0.40-1.04) | 0.083 |
| Diabetes | 1.32 (1.17-1.49) | <0.001 | 1.08 (0.87-1.34) | 0.492 |
| Dyslipidemia | 1.06 (0.94-1.20) | 0.342 | 1.05 (0.90-1.23) | 0.53 |
| Hypertension | 1.23 (1.05-1.44) | 0.009 | 1.13 (0.92-1.39) | 0.234 |
| Potassium-sparing diuretic | 1.21 (0.79-1.78) | 0.36 | 1.14 (0.69-1.84) | 0.597 |
| Corticosteroid | 0.79 (0.64-0.96) | 0.018 | 0.86 (0.67-1.09) | 0.213 |
| Alpha-/beta-blocker | 1.00 (0.82-1.21) | 0.992 | 0.92 (0.73-1.16) | 0.497 |
| Anticoagulant | 0.68 (0.40-1.08) | 0.124 | 0.77 (0.43-1.30) | 0.349 |
| Antiplatelet | 0.96 (0.83-1.11) | 0.617 | 0.88 (0.73-1.06) | 0.187 |
| Direct vasodilator | 0.95 (0.55-1.55) | 0.852 | 0.54 (0.29-0.97) | 0.048 |
| CCB | 1.40 (1.24-1.59) | <0.001 | 1.02 (0.87-1.21) | 0.766 |
| ESA | 1.31 (1.15-1.49) | <0.001 | 0.88 (0.72-1.08) | 0.231 |
| NSAID | 0.89 (0.59-1.29) | 0.545 | 1.15 (0.73-1.76) | 0.546 |
| Proton-pump inhibitor | 0.90 (0.75-1.09) | 0.288 | 0.96 (0.76-1.20) | 0.703 |
| Loop diuretic | 1.24 (1.08-1.41) | 0.002 | 0.96 (0.81-1.15) | 0.685 |
| Statins | 1.04 (0.92-1.17) | 0.546 | 0.93 (0.80-1.09) | 0.385 |
| Oral hypoglycemic | 1.33 (1.17-1.50) | <0.001 | 1.07 (0.87-1.32) | 0.539 |
| Xanthine oxidase inhibitor | 1.29 (1.14-1.46) | <0.001 | 1.01 (0.86-1.18) | 0.903 |
| Insulin | 1.37 (1.17-1.59) | <0.001 | 1.06 (0.87-1.30) | 0.541 |

Abbreviations: CHF, congestive heart failure; CAD, coronary artery disease; CVA, Cerebrovascular accident; ACEi, Angiotensin-converting enzyme inhibitors; ARB, Angiotensin receptor blocker; CCB, Calcium channel blocker; ESA, Erythropoiesis stimulating agent; CPS, Calcium polystyrene sulfonate; NSAID, Nonsteroidal anti-inflammatory drugs;
